# Supplementary material for: Honey Bee Pathogen Prevalence and Interactions within the Marmara Region of Turkey
Source: Vet Sci. 2022 Oct 17;9(10):573. doi: 10.3390/vetsci9100573 (PMC9610934; doi:10.3390/vetsci9100573)
Supplement: Supplementary file 1 [file vetsci-09-00573-s001.zip › vetsci-1922614-supplementary.pdf]

Table S1. Primers used for molecular disease screenings. SB: stonebrood, CB: chalkbrood, AFB: American foulbrood, CER: *Nosema ceranae*, APIS: *Nosema apis*, RpS5: ribosomal protein S5 of *Apis mellifera*.

**Primer**	**Sequence (5′-3′)**	**Product length (bp)**		**Ref.**
SB-F	GGTAACCAAATCGGTGCTGCTTTC	A. fumigatus	549	[1]
		A. flavus	550	
		A. niger	531	
SB-R	ACCCTCAGTGTAGTGACCCTTGGC	*A. terreus*	564	
		A. clavatus	562	
		A. nidulans	475	
CB-F	TGTCTGTGCGGCTAGGTG	648		[2]
CB-R	CCACTAGAAGTAAATGATGGTTAGA			
AFB-F	TTCGGGAGACGCCAGGTTA	131		[3]
AFB-R	CTTTCATGACTTCTTCATGCGAAG			
CER-F	CGGCGACGATGTGATATGAAAATATTAA	218		[4]
CER-R	CCCGGTCATTCTCAAACAAAAAACCG			
APIS-F	GGGGGCATGTCTTTGACGTACTATGTA	321		
APIS-R	GGGGGGCGTTTAAAATGTGAAACAACTATG			
RpS5-F	AATTATTTGGTCGCTGGAATTG	115		[5]
RpS5-R	TAACGTCCAGCAGAATGTGGTA			

Table S2. Comparisons in residual deviance between models containing no interactions, ABPV-BQCV interaction, DWV-KV-VDV1 interactions, and ABPV-BQCV and DWV-KV-VDV1 interactions using the likelihood ratio test for each disease. $\chi 2$: Chi-square statistic, Df: degrees of freedom.

**Response variable**	**Interaction effects**	**Deviance**	χ^2	**Df**	***p*-value**
Varroa	NA	789.67			
	ABPV-BQCV	788.80	0.8620	1	0.35
	DWV-KV-VDV1	784.88	3.9198	2	0.14
	All	783.78	1.1074	1	0.29
SB	NA	117.92			
	ABPV-BQCV	117.19	0.73	1	0.39
	DWV-KV-VDV1	113.87	3.32	2	0.19
	All	113.09	0.78	1	0.38
Nosema	NA	156.23			
	ABPV-BQCV	156.14	0.09	1	0.77
	DWV-KV-VDV1	153.89	2.26	2	0.32
	All	153.74	0.15	1	0.70
AFB	NA	134.45			
	ABPV-BQCV	133.85	0.60	1	0.44
	DWV-KV-VDV1	131.64	2.21	2	0.33
	All	131.27	0.37	1	0.54
DWV	NA	3625.1			

	KV-VDV1	3606.4	18.67	3	**<0.001**
	All	3606.3	0.07	2	0.79
KV	NA	3407.2			
	ABPV-BQCV	3406.8	0.43	0	NA
	DWV-KV	3393.2	13.60	1	**<0.001**
	All	3393.2	0.02	1	0.88
VDV1	NA	3631.6			
	ABPV-BQCV	3631.6	0	0	NA
	DWV-KV	3614.5	17.16	1	**<0.001**
	All	3611.1	20.53	2	**<0.001**
ABPV	NA	2194.0			
	DWV-KV-VDV1	2187.9	6.08	3	0.11
BQCV	NA	3145.0			
	DWV-KV-VDV1	3144.4	0.65	3	0.88

References

1. Nasri, T.; Hedayati, M.T.; Abastabar, M.; Pasqualotto, A.C.; Armaki, M.T.; Hoseinnejad, A.; Nabili, M. PCR-RFLP on β-Tubulin Gene for Rapid Identification of the Most Clinically Important Species of Aspergillus. *J. Microbiol. Methods* **2015**, *117*, 144–147, https://doi.org/10.1016/j.mimet.2015.08.007.
2. James, R.R.; Skinner, J.S. PCR Diagnostic Methods for Ascosphaera Infections in Bees. *J. Invertebr. Pathol.* **2005**, *90*, 98–103, https://doi.org/10.1016/j.jip.2005.08.004.
3. Rossi, F.; Amadoro, C.; Ruberto, A.; Ricchiuti, L. Evaluation of Quantitative PCR (QPCR) Paenibacillus Larvae Targeted Assays and Definition of Optimal Conditions for Its Detection/Quantification in Honey and Hive Debris. *Insects* **2018**, *9*, 165, https://doi.org/10.3390/insects9040165.
4. Hamiduzzaman, M.Md.; Guzman-Novoa, E.; Goodwin, P.H. A Multiplex PCR Assay to Diagnose and Quantify Nosema Infections in Honey Bees (Apis Mellifera). *J. Invertebr. Pathol.* **2010**, *105*, 151–155, https://doi.org/10.1016/j.jip.2010.06.001.

5. Thompson, G.J.; Yockey, H.; Lim, J.; Oldroyd, B.P. Experimental Manipulation of Ovary Activation and Gene Expression in Honey Bee (Apis Mellifera) Queens and Workers: Testing Hypotheses of Reproductive Regulation. *J. Exp. Zool. Part Ecol. Genet. Physiol.* **2007**, *307A*, 600–610, https://doi.org/10.1002/jez.415.
